# Bibliometric analysis: a study of the microenvironment in cervical cancer (2000-2024)

**DOI:** 10.3389/fonc.2025.1508173

**Published:** 2025-02-27

**Authors:** Yun-Tao Zhang, Yan-Ni Wei, Chen-Chen Liu, Mai-Qing Yang

**Affiliations:** ^1^ Department of Obstetrics, Changyi People’s Hospital, Changyi, Shandong, China; ^2^ Faculty of Health Management, Weifang Nursing Vocational College, Weifang, Shandong, China; ^3^ Department of Pathology, Weifang People’s Hospital (First Affiliated Hospital of Shandong Second Medical University), Weifang, Shandong, China

**Keywords:** cervical cancer, tumor microenvironment, bibliometric analysis, VOSviewer, CiteSpace

## Abstract

**Objective:**

The incidence of cervical cancer has increased in recent years. The tumor microenvironment (TME) is the local biological environment involved in tumor occurrence and development. This study aimed to conduct a comprehensive analysis of the global research on the TME in cervical cancer (CC), providing a knowledge framework in this field from a holistic and systematic perspective based on a bibliometric analysis.

**Methods:**

Studies focusing on the TME in cervical cancer were searched using the Web of Science Core Collection database. The annual output, cooperation, hotspots, research status, and development trends in this field were analyzed using bibliometric softwares (VOSviewer and CiteSpace).

**Results:**

A total of 1,057 articles published between 2000 and 2024 were selected. The number of publications and citations has recently increased. Cooperation network analysis indicated that China holds the foremost position in research on the TME in cervical cancer with the highest volume of publications, thus exerting the greatest influence. Fudan University had the highest output. Frontiers in Oncology showed the highest degree of productivity in this field. Rofstad, Einar K. made the most article contributions and was the most co-cited author. Four clusters were obtained after a cluster analysis of the keywords: TME, cervical cancer, immunotherapy, and prognosis. Immunotherapy, human papillomavirus, and biomarkers were relatively recent keywords that attracted increasing attention from researchers.

**Discussion:**

This bibliometric analysis provides a data-based and objective introduction to the TME of cervical cancer, and offers readers a valuable reference for future research.

**Conclusions:**

Comprehensive research in this field was mainly distributed in the TME of cervical cancer through the analysis of keywords and documents. Sufficient evidence supports mechanism research and application exploration. Further research should explore new topics related to the TME of cervical cancer.

## 1 Introduction

Cervical cancer (CC) is a significant global public health issue. It is the fourth most common cancer among women and a leading cause of cancer-related morbidity and mortality worldwide. Over 660,000 women are diagnosed with CC, and over 348,000 die every year (1, 2). The high-risk age for CC is 35-45 years old (3, 4). Approximately 85% of CC-related fatalities occur in low-income or developing countries, where the survival rate is significantly lower than that in affluent nations (5). According GLOBOCAN 2020 database to estimate the age-specific and age-standardized incidence and mortality rates of CC per 100 000 women-years for 185 countries (1). Regions with the highest occurrence rates of CC include the Caribbean, Central American, South American, Sub-Saharan African, and South Asian countries. Even in developed countries like the United States (1). CC incidence varied by at least 10 times between regions, it was high in Malawi and Zambia in Africa. Mortality rates ranged from 1.0 (0.8–1.2) in Switzerland to 55.7 (47.7–63.7) in Eswatini (1). Cervical carcinomas encompass various histological subtypes, with squamous cell carcinoma being the main type (6). Most CC cases are triggered by human papillomavirus (HPV) infection (7). Although early screening for CC has been done well, which can detect and treat CC early, the recurrence and metastasis of CC are still difficult to treat.

Tumor microenvironment (TME) denotes the non-cancerous cells and components present in the tumor. In addition to malignant cells, adipocytes, fibroblasts, tumor vasculature, lymphocytes, dendritic cells, and cancer-associated fibroblasts are present in the TME (8, 9). Constant interactions between tumor cells and the TME play a decisive role in tumor development, progression, and response to therapies. The TME has attracted significant research and clinical interest as a therapeutic target for cancer (8, 10, 11). Targeting and manipulating cells and factors in the TME during cancer treatment can help control malignancies and achieve positive health outcomes (12).

In recent years, several studies have been conducted on the TME of CC (13, 14). An increasing number of researchers are focusing on this field. A systematic and holistic literature review will help better understand the current research situation and select research directions. The Web of Science Core Collection (WOSCC) is a widely recognized comprehensive academic literature database that includes references cited in papers and compiles unique citation indices based on cited authors, sources, and publication years (15). Many studies have used this database as a source for bibliometric analyses. Bibliometrics is the analysis of publications using statistics to describe or display the relationships among published works (16, 17). In this study, we used bibliometric analysis to sort and analyze the annual output, cooperation, hotspots, research structure, and development trends in this field from a holistic and systematic perspective.

## 2 Materials and method

### 2.1 Data collection

**Figure 1:**
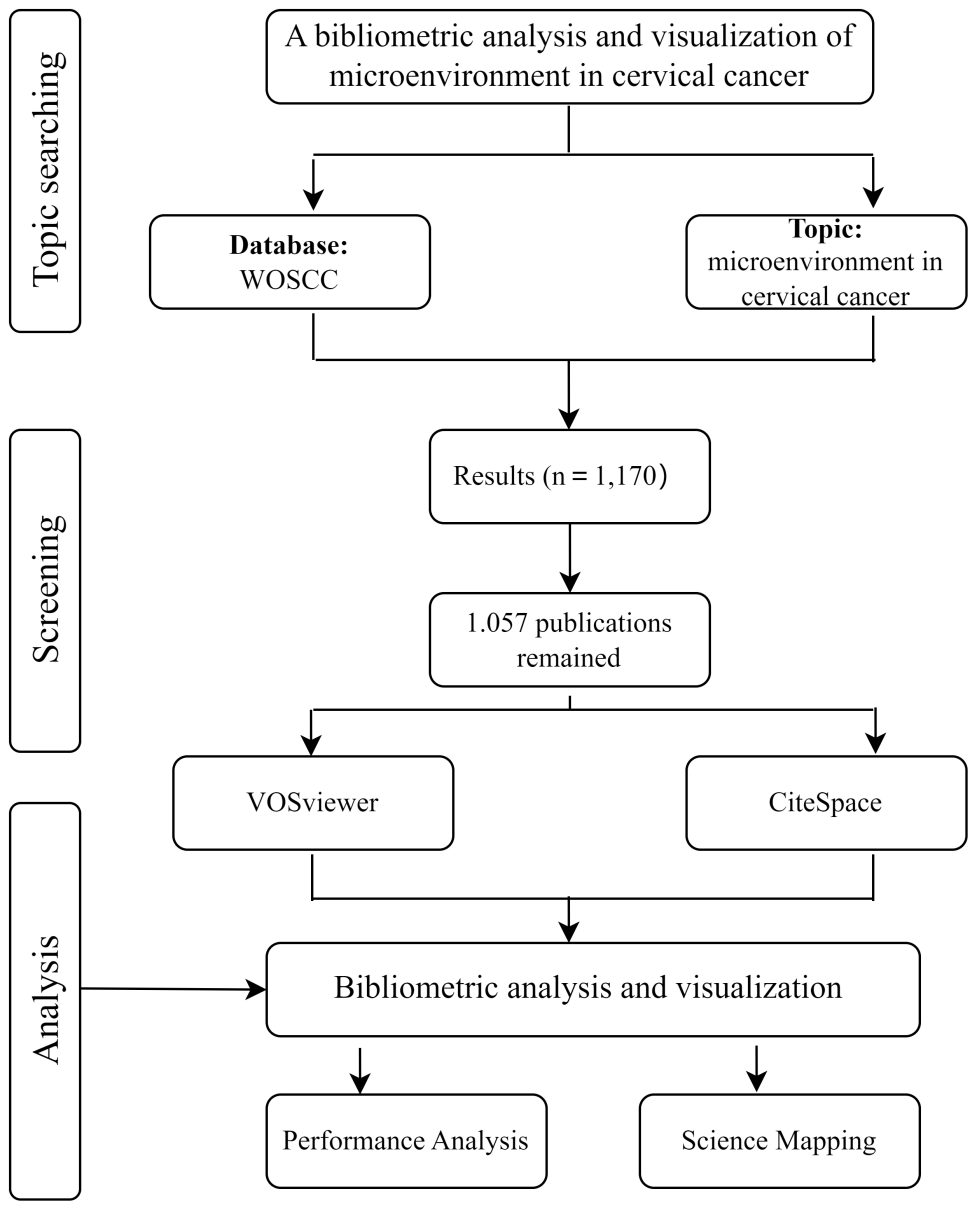
Study flow chart.

We conducted a literature search in the WOSCC (https://www.webofscience.com/wos/woscc/basic-search). The search was conducted on September 30, 2024. The publication period for this study was set from 2000 to 2024. The search terms were presented as follows: Topic = “microenvironment” AND Topic= “cervical cancer” OR “cervical carcinoma” OR “cervical neoplasm”. After the preliminary search, screened the publications based on the following inclusion criteria (1) the publication time span was set from January 1, 2000 to September 30, 2024 (2), only English-language publications were included (3), the publication type was limited to articles or reviews (4), the publication was related to a study of both CC and the tumor microenvironment. In order to ensure the representativeness of the selected publications, the search results underwent a title and abstract-based filtration process, which excluded irrelevant publications. All documents related to CC and TME were exported in “full records and references” TXT format. VOSviewer and CiteSpace were retrieved and imported for bibliometric analysis (18, 19). This research is an observational study. A comprehensive workflow design for literature screening and data analysis is presented in **Figure 1**.

### 2.2 Data analysis and visualization

VOSviewer and CiteSpace were used for bibliometric analysis. VOSviewer (version 1.6.20, Leiden University, The Netherlands) was used to perform a network visualization analysis of the countries, institutions, journals, and keywords that had published relevant literature. Each color in the graph corresponds to a cluster; the more items contained in the cluster, the closer the color is to red, and the fewer the items contained, the closer it is to blue. The size of a node represents its frequency of occurrence in the relevant literature in that research direction, and the thickness of the connecting lines between nodes represents the frequency of co-occurrence. CiteSpace software (version 6.3. R1, Drexel University, PA) was used to generate keyword burst intensity maps. Keyword emergence refers to the sudden and frequent use of keywords within a certain period that can be used to reflect hot topics and future trends in a research field (20, 21). In this study, variables are expressed as numbers and percentages. No comparisons was made; therefore, no P value was set.

## 3 Results

### 3.1 Analysis of publication quantity and trends

There were 1,170 related research documents, 1,057 pieces of literature related to the involvement of the TME in CC were included in the analysis after selecting the language as English. Among these, there were 834 original research articles and 223 reviews. **Table 1** illustrates the annual distribution of these articles. During the initial phase (from 2000 to 2010), very few articles were published. From 2011 to 2020, there was a steady increase in publication volume. The subsequent phase, from 2021 to 2024, was characterized by a significant surge in research activity, reflecting a heightened focus on this area.

**Table 1:** Annual distribution of the number of papers (2000-2024).

Year	Papers	% of 1,057
2024	161	15.23%
2023	166	15.70%
2022	176	16.65%
2021	115	10.88%
2020	84	7.95%
2019	60	5.68%
2018	37	3.50%
2017	44	4.16%
2016	34	3.22%
2015	30	2.84%
2014	28	2.65%
2013	25	2.37%
2012	24	2.27%
2011	16	1.51%
2010	12	1.14%
2009	12	1.14%
2008	8	0.76%
2007	7	0.66%
2006	5	0.47%
2005	3	0.28%
2004	1	0.09%
2003	2	0.19%
2002	4	0.38%
2001	1	0.09%
2000	2	0.19%

### 3.2 Distribution of countries and institutions

#### 3.2.1 Contributions of countries

**Figure 2:**
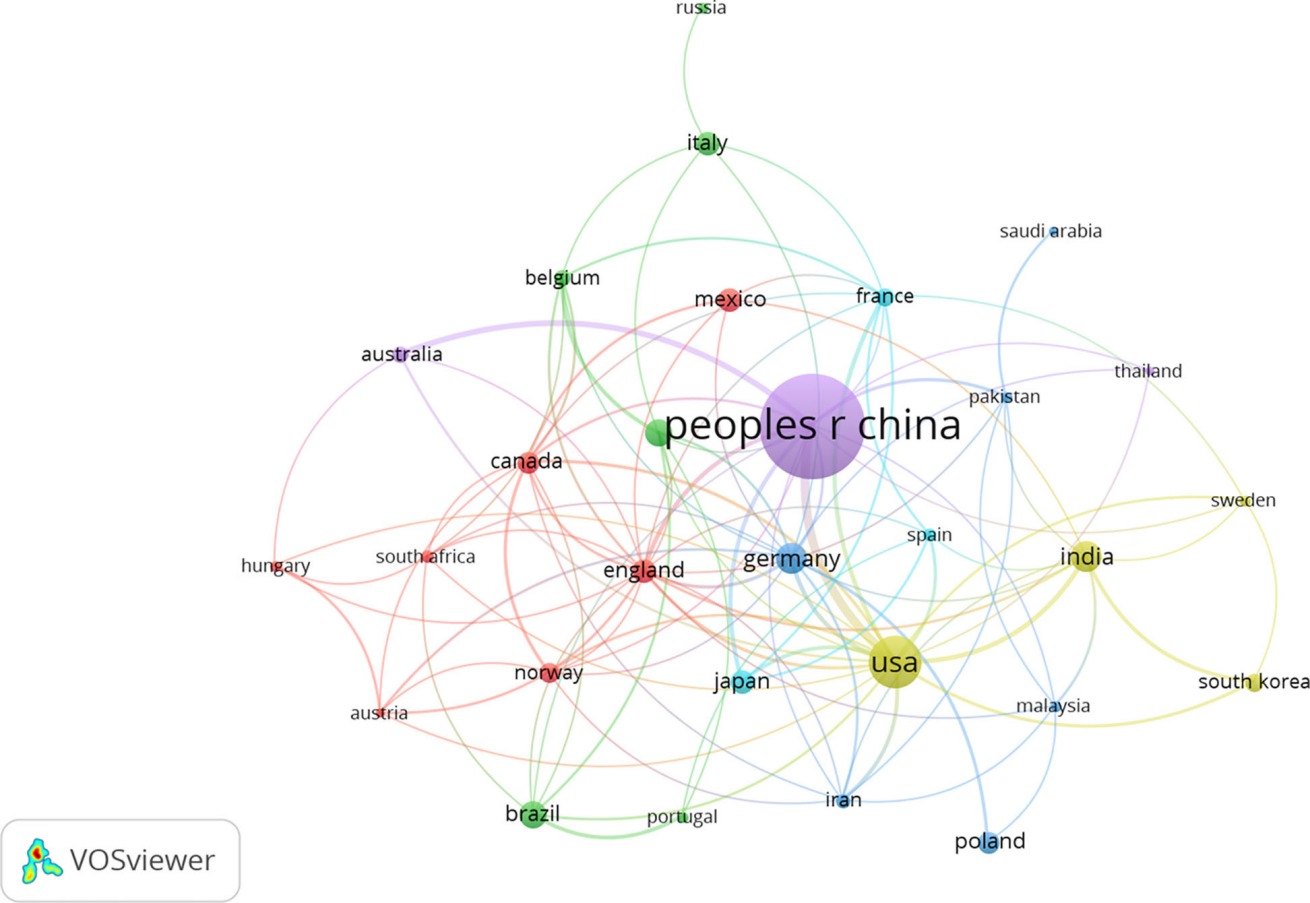
Network visualization map of co-citations of countries. The larger the circle node in the figure, the more papers are published. The node connection represents the association strength; the thicker the connection, the more times the two countries cooperated to publish papers. The node colors represent different clusters.

We used VOSviewer software to analyze the data and generate national visualization maps (**Figure 2**). Results showed top 5 countries for the number of papers published in this field were China (539, 50.99%), the United States (143, 13.53%), Germany (49, 4.64%), India (49, 4.64%) and Brazil (39, 3.69%); top 5 centrally ranked countries were China, the United States, England, Germany, and India (**Table 2**, **Figure 2**). These results suggested that China holds a leading position in this field of research.

**Table 2:** The top 10 countries with the most reviews and total citations.

Number	Countries	Document	Citations
1	China	539	8,000
2	The United States	143	7,650
3	Germany	49	1,346
4	India	49	826
5	Brazil	39	1,111
6	Netherlands	38	1,705
7	Mexico	31	932
8	Japan	30	824
9	England	28	1,671
10	Italy	28	952

#### 3.2.2 Contributions of institutions

**Figure 3:**
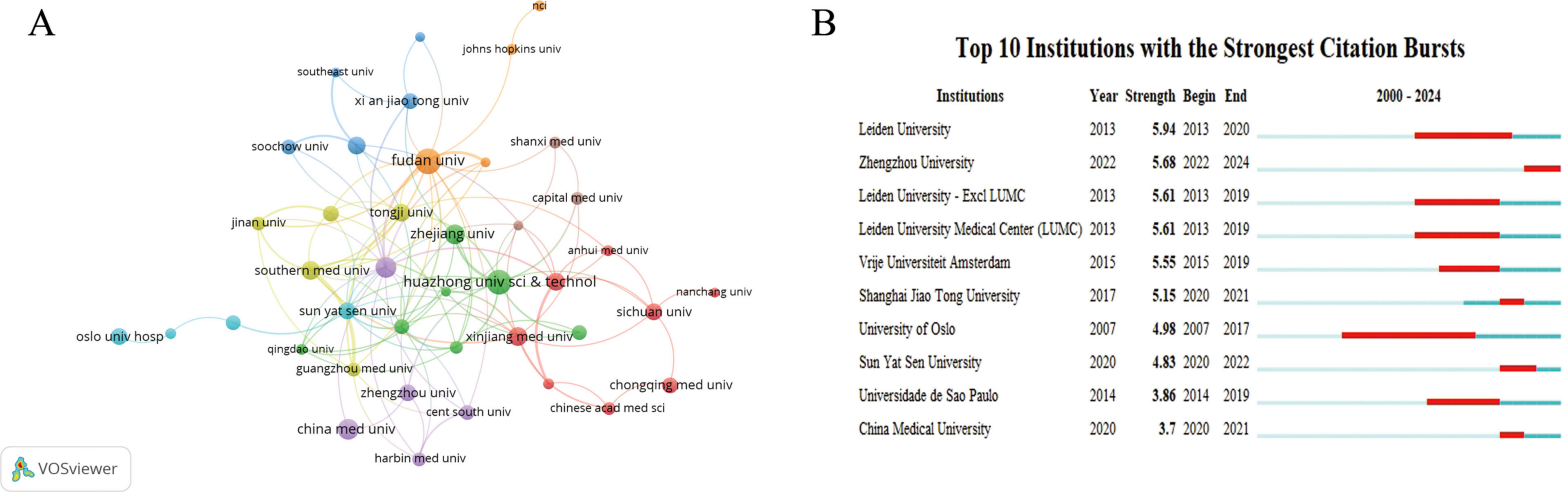
Visualization of institutions. **(A)** Network visualization map of co-citations of institutions. **(B)** Top 10 institutions with the strongest citation bursts.

The field of TME in CC attracted the attention of 1,403 academic institutions. **Figure 3A**, generated using VOSviewer, shows an institutional visualization map. Fudan University was highlighted as the foremost contributor with 40 publications, and other prominent institutions include Huazhong University of Science Technology, Shanghai Jiaotong University, China Medical University, and Zhejiang University (**Table 3**). The institutional analysis showed that the institutions that have published many papers and those with more collaborations were mainly from China (**Figure 3A**). **Figure 3B** lists the top 10 cited institutions, ranked by the duration of their citation bursts, highlighting the significant impact of these institutions.

**Table 3:** The top 15 institutions with the most reviews and total citations.

Number	Institutions	Document	Citations
1	Fudan university	40	695
2	Huazhong university of science technology	36	399
3	Shanghai Jiaotong university	25	449
4	China medical university	25	258
5	Zhejiang university	23	343
6	Xinjiang medical university	22	220
7	Southern medical university	21	593
8	Shandong university	20	383
9	Nanjing medical university	20	117
10	Leiden university	19	1,049
11	Tongji university	19	511
12	Sun yat sen university	18	667
13	Zhengzhou university	18	98
14	Oslo university hospital	17	411
15	Sichuan university	17	371

### 3.3 Analysis of authors

**Figure 4:**
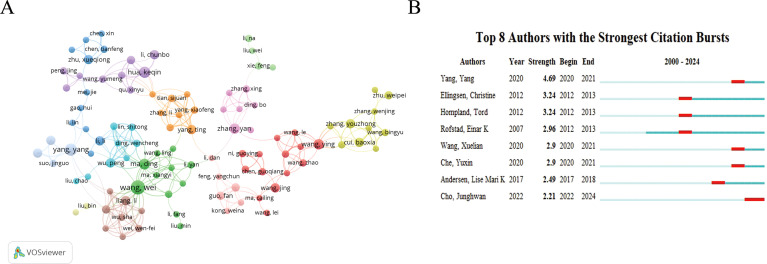
Visualization of authors. **(A)** Network visualization map of co-citations of authors. **(B)** Top 8 authors with the strongest citation bursts.

Examining the seminal work of impactful authors in a field provides a way to comprehend classical theories. In total, 6,279 authors contributed 1,057 publications, yielding a co-authorship index of 5.94. **Table 4** lists the authors who published eight or more research articles with more than 100 citations. The results showed that Rofstad, Einar K. from Oslo University Hospital in Norway, Wang Wei from Sun Yat-sen University in China, and Jordanova Ekaterina S. from Leiden University Medical Center in the Netherlands had the most prolific output and citations. The author co-citation visualization map and top 8 cited authors (**Figure 4**) show that authors are more densely cross-cited and have a significant impact.

**Table 4:** The top authors with the most reviews and total citations.

Number	Authors	Document	Citations
1	Rofstad, einar k.	16	427
2	Wang,wei	14	625
3	Jordanova, Ekaterina s.	12	597
4	Yang,yang	10	202
5	Hua, keqin	9	167
6	Weiss-steider, benny	9	129
7	Gaustad, jon-vidar	8	171
8	Ma,ding	8	142

### 3.4 Analysis of journals

The field of TME in CC attracted the attention of 404 journals. **Table 5** lists the top 15 journals in terms of their publications in this field. Frontiers in Oncology was the most prolific journal, with 45 articles, followed by Frontiers in Immunology (30), Cancers (29), Scientific Reports (21), and International Journal of Molecular Sciences (20). Frontiers in Oncology had the highest citation rate (659 citations). These results indicate the high quality of articles published in these journals and their high scientific value and impact.

**Table 5:** The top 15 journals with the most reviews and total citations.

Number	Journals	Document	Citations
1	Frontiers in oncology	45	659
2	Frontiers in immunology	30	471
3	Cancers	29	341
4	Scientific reports	21	767
5	International journal of molecular sciences	20	412
6	Frontiers in genetics	16	115
7	PLOS ONE	15	562
8	BMC Cancer	14	465
9	Oncoimmunology	12	896
10	Gynecologic oncology	12	542
11	Oncology letters	12	170
12	Heliyon	12	23
13	Journal for immunotherapy of cancer	11	273
14	Cells	11	119
15	Cancer cell international	10	155

### 3.5 Key topics of research hotspots

Keywords condense the core and essence of a paper. Keyword analysis is not only the most effective way to discover research hotspots in a certain scientific field but also to understand the investigation direction of the issues of concern in the topic.

#### 3.5.1 Analysis of clusters and co-occurrence of keywords

**Figure 5:**
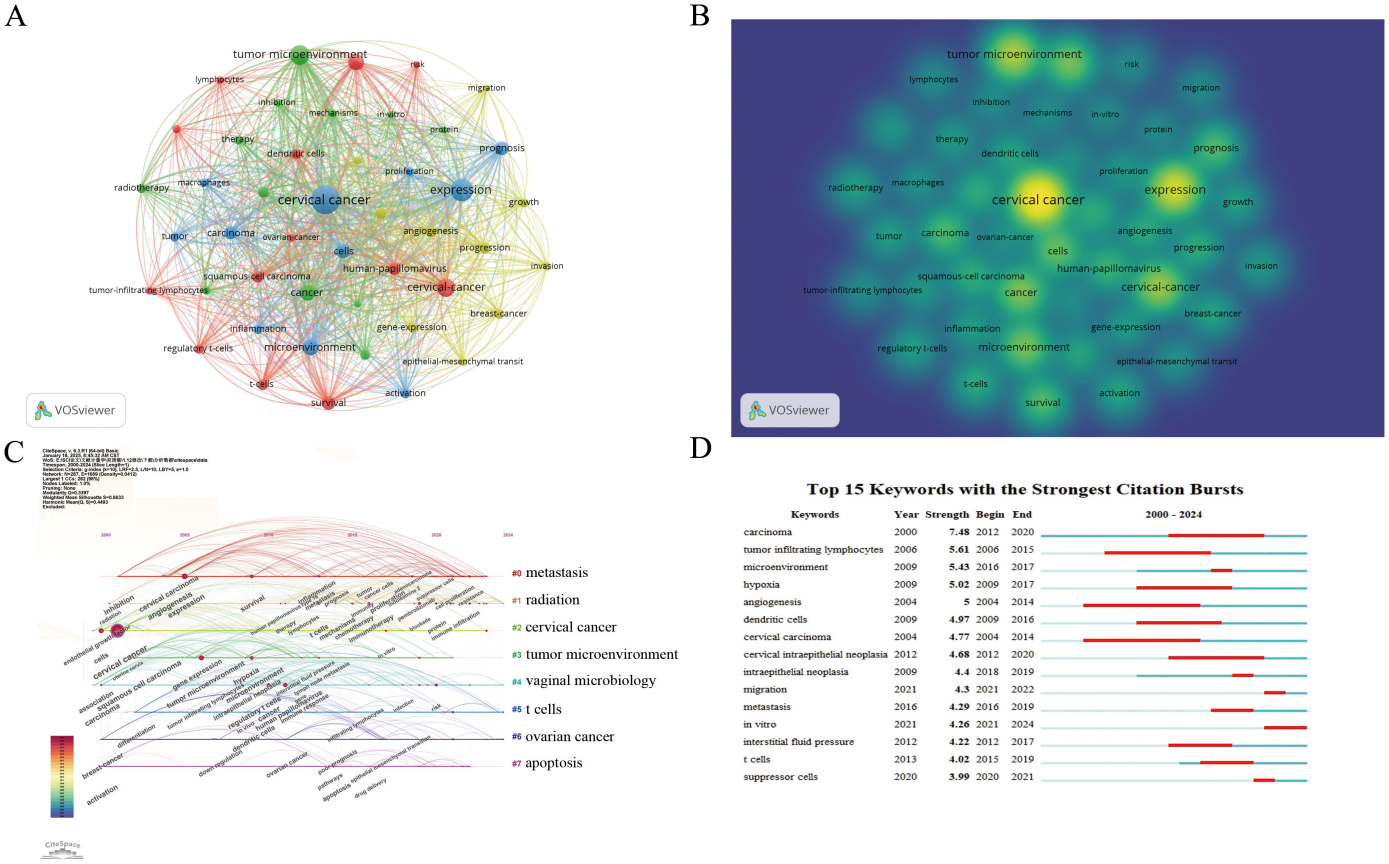
Visualization of keywords. **(A)** Network visualization map of co-citations of keywords. **(B)** Analysis results of hotspot and topic migration in the field of tumor microenvironment in cervical cancer. **(C)** Keyword clustering timeline graph. **(D)** Top 15 keywords with the strongest citation bursts.

VOSviewer was used to draw a keyword co-occurrence network visualization of the 1,057 articles. A total of 4,558 keywords were collected for this study. Based on the link strength of keyword co-occurrence, we selected 46 key keywords with a frequency of occurrence ≥ 30 times for visualization and divided the network into 4 clusters. The concept of different research directions on a topic was proposed through statistical analysis of keywords in different parts of the paper. Cluster analysis of these keywords provided insights into the knowledge structure in this field. Clusters 1 (red) and 2 (green) were the largest with 13 and 12 terms, respectively. The main themes in Cluster 1 (red) were CC, dendritic cells, HPV, immunotherapy, survival, and t-cells. Cluster 2 (green) was mainly associated with cancer, apoptosis, chemotherapy, hypoxia, radiotherapy. Cluster 3 (blue) focused on activation, expression, prognosis, tumor, and inflammation. Cluster 4 (yellow) was related to angiogenesis, growth, metastasis, migration (**Figures 5A, B**).

#### 3.5.2 Burst detection and overlay visualization of keywords

Keyword burst detection facilitates swift identification of emerging research hotspots within a specific field. **Figures 5C, D** show the most robust keyword bursts in the TME of CC research between 2000 and 2024. “Carcinoma” was the most robust burst keyword (strength 7.48) from 2012 to 2020, followed by “tumor infiltrating lymphocytes” (strength 5.61, from 2006 to 2015), “microenvironment” (strength 5.43, from 2016 to 2017), “hypoxia” (strength 5.02, from 2009 to 2017), and “angiogenesis” (strength 5, from 2004 to 2014), revealing that these keywords represented the popular research topics in recent years and even in the near future.

## 4 Discussion

### 4.1 Summary of findings

In this study, the WoSCC database was used to compile publications related to the TME and CC. We utilized VOSviewer and CiteSpace to visualize the spatial and temporal distributions of literature, keywords, publication citations, and co-citations, providing a comprehensive overview of the research landscape. Furthermore, these tools were used to elucidate the current status, hotspots, and trends in research on the TME and CC. This study aimed to provide a valuable reference for researchers in the field of CC.

In this study, 1,057 articles published between 2000 and 2024 were selected. The results indicated that the annual number of publications has been increasing. The year 2012 was the turning point of the annual growth of publications and total annual citations, while 2021 was the vital time for further growth. These were two important periods. Many researchers have devoted themselves to this field, and many journals have begun to pay attention to it. In this research field, the top ten countries accounted for 92.15% of the total publications. China and the United States had the deepest academic accumulation and greatest influence in this field. China demonstrated the highest level of international collaboration and had the highest publication count. Our results showed that China played a leading role, with 8,000 citations in 539 articles. China also had the top ten most productive research organizations, with Fudan University being the most productive institution worldwide. Three of the top eight authors in this field were Chinese: Wang Wei, Yang Yang, and Hua Keqin. These findings highlight the significant contributions and potential scientific innovations of Chinese efforts in the TME of CC research. Although China ranked first in terms of total publication count, the average number of citations per article was only 14.84. The average number of citations per article in the United States was 53.50. This indicates that China still has a long way to go in this field.

Approximately a half of the affiliations had ≤ five articles. This indicated that most affiliations in this field have not made in-depth investments and only a few affiliations have conducted continuous research. Research competitiveness can be improved through international teamwork, suggesting that it is important to seek extensive collaboration among institutions. There were many journals concerned with this field. The development of Frontier serial journals in this field showed vigorous momentum. In particular, Frontiers in Oncology was the most productive. Support was provided for the study of the TME in CC. Current research on the TME in CC is mainly published in the fields of oncology, biology, genetics, immunology, and medicine related journals. This shows that the research is relatively extensive and in the development stage from basic to clinical research.

### 4.2 Research hotspots and frontier exploration in the TME of cervical cancer field

Keywords are a summary of the themes in the literature. The frequency with which keywords appear in the literature can reveal research hotspots in the field. The analysis of keyword co-occurrence can indicate the study category and research hotspots in the field and display discovery trends. Researchers have explored multiple directions of the keyword co-occurrence network and changes in high-frequency keywords over the years. “Carcinoma” was the most robust burst keyword (strength 7.48) from 2012 to 2020, followed by “tumor infiltrating lymphocytes” (strength 5.61, from 2006 to 2015), “microenvironment” (strength 5.43, from 2016 to 2017), “hypoxia” (strength 5.02, from 2009 to 2017), and “angiogenesis” (strength 5, from 2004 to 2014), revealing that the research boom had begun since 2000 and continues to this day. The research structure in this field could be summarized using a keyword co-occurrence network. The first part aimed to determine the relationship between the TME and CC. Then there was the mechanism research, such as “human papillomavirus”, “regulatory t-cells”, and “dendritic cells”. In addition, there was also practical application research, such as prognosis and therapy.

### 4.3 Relationship between TME and cervical cancer

The major risk factors associated with CC include high-risk HPV (hrHPV) infection, age, smoking, childbirth, use of oral contraception, and diet (22–25). Cervical carcinoma arises from the normal cervical epithelium through progressive development, in which hrHPV infection plays a major causative role. The hrHPV infection of the cervical epithelium results in host genome alterations, and the imbalance and instability caused by various hrHPV-derived oncogenic factors in the host genome of cervical epithelial cells drive neoplastic progression (23, 26–28).

Surgical resection and concurrent chemoradiotherapy is the standard-of-care treatment for locally advanced CC (29). The survival of CC patients was lower. Higher stage and tumor size led to shorter survival. The histopathology and type of treatment in comparable stages did not have any significant impact on survival (30). Despite advances in standard therapies, patients with recurrent metastatic CC face a poor prognosis and limited treatment options. Novel treatment strategies are emerging to combat the limited effectiveness of chemotherapy, such as immunotherapy, biomarker-driven personalized therapies (31).

The TME includes noncancerous cells and components present in the tumor, including molecules produced and released by them. Constant interactions between tumor cells and the TME play important roles in tumor initiation, progression, metastasis, and therapy (8, 11, 12, 32, 33). Immunotherapies including immune checkpoint inhibitors and antigen receptor cells have revolutionized cancer treatment (34, 35). Over the past few years, immunotherapy has been used as a therapeutic strategy for clinical oncology. Immunotherapy has emerged as the standard of care for many common cancer types (36). This remarkable increase is largely due to the development of novel checkpoint inhibitors, specifically antibodies targeting programmed cell death 1(PD1) and programmed cell death 1 ligand 1(PDL1). The two main pathways that are specifically targeted in clinical practice are cytotoxic T-lymphocyte antigen-4 and PD 1 that showed potent immune-modulatory effects through their function as negative regulators of T cell activation (37–40). A positive response to immunotherapy usually depends on the interaction between tumor cells and immune regulation within the TME, which plays an important role in suppressing or enhancing immune responses, adoptive cell therapy with tumor-infiltrating lymphocytes has achieved remarkable clinical efficacy in CC (41–43). Immune checkpoint inhibitors have revolutionized the treatment of CC, and promising data are emerging from early phase trials of novel immunotherapeutic approaches, such as HPV therapeutic vaccines (44–48). The early region (E) oncoproteins of HPV are associated with the pathogenesis and contribute to the progression of cancer, inhibition of the activity of E6 and E7 oncoproteins may be a better selective target to delay the progression of CC (28).

### 4.4 Future development

There have been numerous studies on the TME in CC to build a solid foundation for this field; however, it is still at the superficial level at present, and thus researchers should pay more attention to the in-depth mechanism.

## 5 Limitations

This study had some limitations. It only searched the WOSCC database for relevant literature, which may have overlooked relevant studies in other databases and important findings from earlier studies.

## 6 Conclusions

This was the first study to systematically analyze the literature on the TME of CC using a bibliometric approach. Compared to traditional reviews, it provides original and objective insights for research on TME CC-related topics and a valuable reference for further research. This is more credible when two bibliometric tools (VOSviewer and CiteSpace) are used simultaneously for the analysis.

## Data Availability

The original contributions presented in the study are included in the article/supplementary material, further inquiries can be directed to the corresponding author.
